# Evaluating Acute Polytrauma Rehabilitation: A Literature Review With Insights From the UK Major Trauma Network

**DOI:** 10.7759/cureus.99644

**Published:** 2025-12-19

**Authors:** Zabrang Ishaku

**Affiliations:** 1 Trauma and Orthopaedics, Queen Mary University, London, GBR; 2 Trauma and Orthopaedics, Barts Health NHS Trust, London, GBR

**Keywords:** acute polytrauma rehabilitation, early mobilisation, functional outcomes, major trauma, multidisciplinary care, rehabilitation challenges, return to work, trauma systems (uk)

## Abstract

Major trauma is a leading cause of disability in young adults, and modern trauma systems increasingly recognise that survival alone is insufficient; functional recovery and quality of life are essential outcomes. This review evaluates the benefits, challenges, and future development needs of integrating acute polytrauma rehabilitation (APR) into trauma systems, with reference to London’s Major Trauma System. It synthesises contemporary evidence from systematic reviews, clinical audits, the European and UK trauma guidelines, and observational studies addressing APR timing, delivery, outcomes, and system-level implementation. The review demonstrates that APR reduces complications associated with prolonged immobilisation, improves mobility and functional independence, enhances return-to-work outcomes, and may shorten hospital length of stay, which contributes to significant long-term cost savings through improved patient independence and reduced reliance on ongoing health and social care. Future directions should prioritise expanding acute and post-acute rehabilitation capacity, supported by coordinated referral pathways, and strengthening community follow-up services. These measures are essential to providing comprehensive, holistic recovery support, ensuring that trauma survivors not only live but also regain meaningful independence and quality of life.

## Introduction and background

Polytrauma refers to multiple life-threatening injuries, typically defined by an Injury Severity Score ≥16 [1]. Major trauma is a leading cause of disability in young populations, and modern trauma systems now recognise that survival alone is not the endpoint - functional recovery and quality of life (QOL) are equally critical [2]. Acute polytrauma rehabilitation (APR) refers to the coordinated, multidisciplinary delivery of rehabilitation interventions - such as physiotherapy (PT), occupational therapy (OT), psychological support, and early mobilisation commencing during the acute phase of trauma management. It refers to the period from initial haemodynamic stabilisation through early inpatient care, typically encompassing the intensive care unit (ICU) and acute surgical wards, and usually extending from the first 24-72 hours post injury until transfer to step-down or specialist rehabilitation services [3]. The UK Major Trauma Network (MTN) comprises four major trauma centres (MTCs) in London linked to smaller trauma units (TUs), with coordinated pre-hospital care, acute care, and post-hospital rehabilitation services that work with social and community services to deliver the right treatment at the right time [4].

This review synthesises contemporary evidence on APR and provides insights from the London trauma system. Objectives were to critically appraise the benefits and challenges of APR; assess system-level implementation, including economic considerations; and examine the London MTN as a case study to identify lessons and development priorities. It uses London's MTS as a focal case study due to its maturity and comprehensive trauma networks, which provide a concentrated body of UK-relevant evidence. Insights from this system are considered transferable to other high-income trauma networks facing similar pressures in critical care capacity workforce allocation and rehabilitation access, while acknowledging contextual limitations. There is substantial heterogeneity in injury patterns, comorbidities, and rehabilitation needs among polytrauma patients, and this variability influences both individual recovery trajectories and the feasibility of universal system-level recommendations.

## Review

Benefits

APR confers numerous benefits at the patient and system levels. It can prevent complications associated with prolonged immobilisation and critical illness. For example, early mobilisation and physiotherapy in the intensive care unit (ICU) reduce the risk of ICU-acquired weakness, joint contractures, thromboembolism, pressure ulcers, atelectasis, pneumonia, and delirium [2,5]. Mitigating these complications may translate into shorter ICU and hospital length of stay (LOS), although reductions in LOS are not uniformly demonstrated across all trauma populations and often appear most clearly in specific cohorts rather than unselected polytrauma samples [6].

It also promotes better functional outcomes and return to independence. Active engagement in PT, OT, and other modalities soon after injury has been associated with faster regain of mobility and self-care ability by discharge [7]. However, functional gains are influenced by potential confounders, including injury severity, baseline frailty, premorbid status, and rehabilitation timing/intensity, which qualify the interpretation of early rehabilitation effects.

In the longer term, an interdisciplinary rehabilitation approach - including physical, cognitive, and psychosocial interventions - offers the best chance to maximise recovery. Multidisciplinary rehabilitation is considered to “offer the best way to improve trauma patient outcomes” by addressing the whole person rather than just injuries in isolation [8]. Evidence linking early rehabilitation to return-to-work (RTW) is promising but methodologically variable, with many studies being observational, using small or selected samples, heterogeneous interventions, and limited follow-up. Although literature is still emerging, clinical experience suggests that patients who receive coordinated rehabilitation early are more likely to regain functional independence, RTW, and integrate into society than those whose rehabilitation is delayed [9]. Early engagement of therapy services can improve these trajectories. For example, a systematic review on vocational rehabilitation intervention to support RTW following traumatic injury found early intervention to be among the effective mechanisms for addressing unmet needs of trauma survivors relating to RTW [10].

Another benefit of integrating rehabilitation into trauma systems is continuity and coordinated care. In an inclusive trauma system, the patient’s journey spans pre-hospital care, acute care, and post-acute rehabilitation [11]. By embedding rehabilitation professionals (PTs, OTs, rehabilitation medicine physicians, psychologists, etc.) within the acute trauma team, there is a smoother handover through each phase. Goals and care plans are shared among surgeons, intensivists, and therapists from the outset, leading to a unified approach. Ideally, “clinical collaboration between team members ensures successful integration of medical, rehabilitative, psychosocial, and financial resources across specialties” [5]. This means, for instance, that, while life-saving surgical and critical care is ongoing, plans for mobilisation, neurological stimulation, delirium prevention, and discharge needs are made in parallel. Such a model can improve efficiency, avoiding delays that occur when rehabilitation is a separate, sequential step.

Early involvement of rehabilitation also provides psychological support and patient-centred focus during what can be a disorienting acute hospital stay, improving patient satisfaction and mental well-being. Engaging patients in goal-directed activity gives a sense of progress and can reduce trauma-related stress. Indeed, some trials have shown that early psychological interventions alongside acute rehabilitation, although inconclusive in the mid-term, decrease long-term post-traumatic stress disorder (PTSD) and depression rates [12]. Evidence is limited by small samples, variable interventions, and short follow-up, so conclusions should remain cautious.

Finally, integrated acute rehabilitation may yield system-wide cost-benefits. Catastrophic injuries often result in prolonged hospitalisations and long-term care if debilities are not mitigated. The 2019 National Clinical Audit of Specialist Rehabilitation (NCASRI) audit found that timely specialist rehabilitation produced mean net lifetime savings of approximately £500,000 per patient with very severe injuries, translating to hundreds of millions of pounds in national savings through improved patient independence and long-term costs [13]. These estimates apply primarily to high-complexity cohorts within specialist rehabilitation pathways, not universally across trauma populations. However, there remains a notable evidence gap regarding cost impacts specific to the acute rehabilitation phase, as most economic evaluations assess the broader specialist rehabilitation trajectory rather than APR itself. By investing in rehabilitation personnel and acute rehabilitation beds, hospitals can potentially reduce downstream resource use, such as expensive nursing home placements or avoidable complications. While upfront resources are needed, the long-term return on investment in APR is likely high, through both humanistic outcomes (better QOL) and economic outcomes (patients returning to productivity and requiring fewer health/social services).

Challenges

Despite clear benefits, integrating rehabilitation into the acute trauma pathway faces significant challenges. One major challenge is timing and medical acuity - polytrauma patients often have life-threatening injuries that necessitate surgeries, intensive care, and hemodynamic stabilisation, which may limit or complicate early rehabilitation. Determining the optimal timing and intensity of interventions requires judgement and protocol development. Mobilising too early could compromise fractures or surgical repairs, whereas waiting too long risks deconditioning. High-quality evidence defining the optimal timing, intensity, and progression of rehabilitation for specific injury subgroups, such as combined traumatic brain injury and orthopaedic trauma, unstable pelvic fractures, or patients requiring prolonged ventilation, remains sparse [3,14]. This uncertainty contributes to clinical variation, which reflects not only the absence of universally agreed guidelines but also contextual constraints, including surgical precautions, staffing availability, and ICU culture. Mobilising too early may risk compromising fracture fixation, surgical repairs, or neurological recovery, while excessive delay increases deconditioning risk. For example, a patient with multiple orthopaedic injuries and traumatic brain injury presents a dilemma of balancing early mobilisation with neurological rest. This highlights the need for clearer clinical criteria, decision aids, or stratified protocols to support clinicians in balancing surgical safety with rehabilitation goals. In practice, some centres pursue aggressive early rehabilitation, while others adopt more cautious approaches, reflecting differences in expertise, risk tolerance, and organisational capacity rather than guideline absence alone [3,15].

Another fundamental challenge is resource limitation, both in the workforce and dedicated rehabilitation facilities [16]. Integrating rehabilitation into acute trauma care is resource-intensive, requiring coordinated input from physiotherapists, occupational therapists, rehabilitation medicine physicians, psychologists, and specialist nursing staff. Shortages in physiotherapists and occupational therapists staffing most directly limit early mobilisation and functional practice, while deficits in rehabilitation medicine and psychology services constrain goal-setting, cognitive rehabilitation, and psychosocial support.

In the UK, after regional MTNs were established, it was found that “from the outset, networks tried to focus on rehabilitation but lack of facilities and manpower has been a significant issue” [17]. All MTCs are expected to maintain a core rehabilitation team, yet significant shortfalls remain. The NCASRI audit reported that fewer than half of MTNs initially met the recommendation to appoint a full-time consultant in rehabilitation medicine to lead acute trauma rehabilitation services [13]. Such gaps inevitably lead to disjointed rehabilitation efforts. Provision of physiotherapists and occupational therapists in trauma wards frequently falls below demand, particularly during weekends and out-of-hours periods, limiting therapy intensity and continuity of care.

Capacity constraints extend beyond the acute hospital. There is a shortage of specialist inpatient rehabilitation beds and step-down services in many regions [13,14]. These limitations are partly attributable to post-acute rehabilitation pathways and commissioning structures rather than acute hospital capacity alone. As a consequence, patients may experience delayed transfers or premature repatriation without specialist rehabilitation input. Although the resulting "postcode lottery" is more clearly demonstrated through service provision and audit data than through comparative functional outcome studies, regional variation in access to specialist rehabilitation is consistently reported. Finally, variability in commissioning and funding structures across regions contributes to persistent workforce and capacity shortfalls, exacerbating inequities in access to integrated rehabilitation. Together, these acute and post-acute constraints demonstrate that even well-designed clinical protocols cannot succeed without sufficient staffing, infrastructure, and sustained investment.

Trauma rehabilitation in London

London’s MTS, established in 2010, provides a valuable case study of integrating rehabilitation into a mature trauma network [11]. Compared with other UK regions, London’s system is distinctive in its early development, higher patient volumes, and concentration of specialist services, although it shares core organisational principles such as inclusive trauma networks and mandated rehabilitation planning with other MTNs.

The city’s trauma system comprises four MTCs - the Royal London Hospital (RLH), St. Mary’s Hospital, King’s College Hospital, and St. George’s Hospital, each linked with designated local TUs. From the outset, the system was designed to be inclusive, encompassing the entire patient pathway from pre-hospital care through acute treatment to rehabilitation and community reintegration [11,18].

Each MTC maintains a multidisciplinary trauma ward where rehabilitation professionals are embedded; however, the extent and consistency of embedded rehabilitation provision vary in practice due to local workforce and resource constraints rather than a fully uniform model. Rehabilitation input is typically coordinated through weekly multidisciplinary team (MDT) meetings, where patient progress and ongoing plans are reviewed. A key strength of the UK - and particularly London - model is its policy-driven emphasis on rehabilitation, supported by mandated rehabilitation prescriptions at discharge and early involvement of rehabilitation staff [11,19]. Mandated rehabilitation quality indicators include timely assessment by therapy services, documentation of a rehabilitation prescription, and referral to specialist rehabilitation where appropriate, with compliance monitored through regular audit and Trauma Audit and Research Network (TARN) submissions.

Compliance with these indicators is monitored through routine audit and data submission to the TARN, which enables benchmarking and continuous improvement [20]. Structural redesign of the trauma system has been associated with measurable improvements - including reduced mortality, faster access to specialist interventions, and more consistent rehabilitation planning - compared with the pre-2010 model [21]. Despite these strengths, persistent bottlenecks remain, particularly in referral processes and access to specialist rehabilitation beds. NCASRI reports ongoing delays in referral, variability in early specialist rehabilitation decision-making, and insufficient specialist bed capacity for patients with complex injuries [13].

Future needs

Enhancing rehabilitation capacity is a recognized priority highlighted by the NCASRI audit [13]. London’s MTS must invest in greater rehabilitation capacity and streamlined pathways, because demand for post-acute rehabilitation far outstrips supply, leading to delayed transfers and suboptimal therapy for many patients. These delays tie up acute beds and worsen outcomes, as prolonged immobilization makes recovery much harder.

The system should expand dedicated trauma rehabilitation facilities and staff to fix this. Investing in additional specialist beds, including “hyper‑acute” units within MTCs, would allow stable post‑ICU patients to access intensive rehabilitation sooner. At the same time, the rehabilitation workforce must be strengthened. Recruitment of full‑time rehabilitation medicine consultants, physiotherapists, occupational therapists, and psychologists is essential to meet increased capacity. Minimum daily therapy targets should be based on existing evidence - the guidelines for stroke rehabilitation recommend at least 45 minutes of each therapy (PT, OT, and speech and language therapy) five days per week, totalling at least three hours of therapy per day [22]. Evidence to support higher dose targets in trauma populations is limited, and future research should clarify the optimal intensity and sequencing of therapies across injury types. Adopting standardised protocols with flexibility for individual needs can improve care quality while acknowledging current gaps in the evidence.

Operational changes are also required. Implementing a centralised referral and bed‑matching system - potentially building on the TARN database - could reduce wait times by matching patients to available rehabilitation beds. However, such a system would need robust digital infrastructure, data governance, and privacy safeguards. Challenges include integrating data from multiple trusts, ensuring timely updates, and training clinicians to use referral tools. Overly rigid algorithms may inadvertently disadvantage patients with uncommon injuries or complex social needs. In the interim, clear referral pathways and shared communication platforms can improve coordination between acute hospitals and rehabilitation providers.

Beyond increasing capacity, the trauma system must address long‑term psychosocial and vocational rehabilitation. Studies of survivors of critical illness show that approximately 40% of patients experience anxiety or depression and around 20% meet criteria for post‑traumatic stress disorder within 12 months of discharge, although estimates vary widely between cohorts and settings [23]. Mental health needs persist long after hospital discharge, and patients and families often lack access to specialist psychological support. In London, about one‑third of severely injured patients do not return to their pre‑injury work or education level, but RTW rates vary substantially across studies, ranging from roughly 37% to 82% depending on injury severity, psychological morbidity, and socio‑economic context [1]. Comprehensive long‑term support should therefore include routine screening for anxiety, depression, and PTSD; sustained access to trauma‑specialist psychologists; and family‑inclusive counselling. Vocational rehabilitation interventions, such as the ROWTATE programme in England, involve early assessment of job demands, goal setting, employer engagement, and ongoing case coordination by occupational therapists and psychologists; evidence from military and stroke populations suggests potential benefits, but data on polytrauma are limited, and scalability in civilian trauma systems is uncertain [24]. Pilot programmes co‑funded by the health and employment sectors could test the feasibility and effectiveness of vocational rehabilitation in the civilian setting.

Evaluation of these initiatives should be embedded in their implementation. Key indicators might include time from acute stabilisation to rehabilitation admission; proportion of patients accessing hyper‑acute or specialist rehabilitation; adherence to minimum therapy targets; functional independence scores at discharge; rates of return to work or education at six and 12 months; prevalence of anxiety, depression, and PTSD at follow‑up; patient and family satisfaction; and cost‑effectiveness metrics. Collecting and analysing such data will allow stakeholders to identify bottlenecks, justify investments, and iteratively refine rehabilitation pathways.

## Conclusions

APR is an integral component of modern trauma care, but the supporting evidence base remains heterogeneous and dominated by observational studies. Nonetheless, consistent themes emerge: early, multidisciplinary rehabilitation improves function, reduces complications, and increases the likelihood of returning to work; integrated systems yield better continuity; and specialist services can be cost‑effective despite higher upfront costs. These conclusions are likely generalisable to high‑income healthcare systems with organised trauma networks, yet the specific barriers and solutions identified in London may not translate directly to other regions with different resource profiles and governance structures.

Strategic investment should prioritise three areas: (1) infrastructure - expanding specialist and hyper‑acute rehabilitation beds and developing digital referral systems with appropriate data governance; (2) workforce - recruiting and training rehabilitation medicine consultants, therapists, and psychologists to meet recommended therapy dose targets; and (3) long‑term support - embedding psychological services and vocational rehabilitation within trauma pathways. Research is needed to refine the timing and intensity of rehabilitation for different injuries, evaluate workforce models and multidisciplinary team configurations, and measure long‑term outcomes, including quality of life, mental health, and employment. Transparent reporting of outcomes and economic analyses will guide policymakers and commissioners. By combining evidence‑based interventions with coordinated planning and sustained funding, trauma systems like that of London can ensure that survivors not only live but also recover meaningful independence and participation in society.
